# Colonization with extended-spectrum cephalosporin-resistant Enterobacterales (ESCrE) in hospitalized patients in Botswana

**DOI:** 10.1017/ash.2023.336

**Published:** 2023-09-29

**Authors:** Naledi Betsi Mannathoko, Mosepele Mosepele, Robert Gross, Rachel Smith, Ashley Styczynski, Leigh Cressman, Melissa Richard-Greenblatt, Laurel Glaser, Kevin Alby, Anne Jaskowiak, Kgotlaetsile Sewawa, Laura Cowden, Dimpho Otukile, Giacomo Paganotti, Margaret Mokomane, Warren Bilker, Ebbing Lautenbach

## Abstract

**Background:** The epidemiology of extended-spectrum cephalosporin-resistant Enterobacterales (ESCrE) in hospitalized patients in low- and middle-income countries (LMICs) is poorly described. Although risk factors for ESCrE clinical infection have been studied, little is known of the epidemiology of ESCrE colonization. Identifying risk factors for ESCrE colonization, which can predispose to infection, is therefore critical to inform antibiotic resistance reduction strategies. **Methods:** This study was conducted in 3 hospitals located in 3 districts in Botswana. In each hospital, we conducted ongoing surveillance in sequential units hospitalwide. All participants had rectal swabs collected which were inoculated onto chromogenic media followed by confirmatory testing using MALDI-TOF MS and VITEK-2. Data were collected via interview and review of the inpatient medical record on demographics, comorbidities, antibiotic use, healthcare exposures, invasive procedures, travel, animal contact, and food consumption. Participants with ESCrE colonization (cases) were compared to noncolonized participants (controls) using bivariable and multivariable analyses to identify risk factors for ESCrE colonization. **Results:** Enrollment occurred from January 15, 2020, to September 4, 2020, and 469 participants were enrolled. The median age was 42 years (IQR, 31–58) and 320 (68.2%) were female. The median time from hospital admission to date of sampling was 5 days (IQR, 3–12). There were 179 cases and 290 controls (ie, 38.2% of participants were ESCrE colonized). Independent risk factors for ESCrE colonization were a greater number of days on antibiotic, recent healthcare exposure, and tending swine prior to hospitalization. (Table). **Conclusions:** ESCrE colonization among hospitalized patients was common and was associated with several exposures. Our results suggest prior healthcare exposure may be important in driving ESCrE. The strong link to recent antibiotic use highlights the potential role of antibiotic stewardship interventions for prevention. The association with tending swine suggests that animal husbandry practices may play a role in community exposures, resulting in colonization detected at the time of hospital admission. These findings will help to inform future studies assessing strategies to curb further emergence of hospital ESCrE in LMICs.

**Figure:**
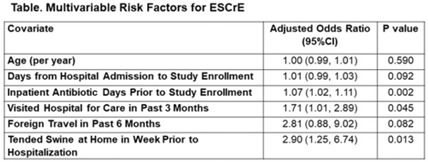

**Disclosures:** None

